# Effectiveness of the Hydrogen Sulfide Test as a Water Quality Indicator for Diarrhea Risk in Rural Bangladesh

**DOI:** 10.4269/ajtmh.17-0387

**Published:** 2017-10-16

**Authors:** Mahfuza Islam, Ayse Ercumen, Abu Mohd Naser, Leanne Unicomb, Mahbubur Rahman, Benjamin F. Arnold, John M. Colford, Jr., Stephen P. Luby

**Affiliations:** 1 Environmental Intervention Unit, Enteric and Respiratory Infections Program, Infectious Disease Division, International Centre for Diarrheal Disease Research, Bangladesh (icddr,b);; 2 Division of Epidemiology, School of Public Health, University of California, Berkeley, California;; 3 Department of Environmental Health Sciences. Rollins School of Public Health, Emory University, Atlanta, Georgia;; 4 Woods Institute for the Environment, Stanford University, Stanford, California

## Abstract

Microbiological water quality is usually assessed by the identification of *Escherichia coli* (*E. coli*), a fecal indicator. The hydrogen sulfide (H_2_S) test is an inexpensive, easy-to-use, and portable alternative field-based water quality test. Our study evaluated the H_2_S test’s effectiveness as a water quality indicator for diarrhea risk. Field workers collected stored drinking water samples for H_2_S analysis and detection of *E. coli* by membrane filtration and measured caregiver-reported diarrhea among children < 5 years in the same households 1 month later. We assessed the association between the H_2_S test (incubated for 24 hours and 48 hours) and diarrhea prevalence, with 2-day and 7-day symptom recall periods (*N* = 1,348). We determined the sensitivity, specificity, and positive and negative predictive value (PPV, NPV) of the H_2_S test compared with *E. coli* (*N* = 525). Controlling for potentially confounding covariates, H_2_S-positive water (at 24 or 48 hours) was not associated with 2-day diarrhea prevalence (24-hour prevalence ratio [PR] = 1.03, 95% confidence interval [CI]: 0.63–1.69; 48-hour PR = 0.89, 95% CI: 0.58–1.38) or 7-day diarrhea prevalence (24-hour PR = 1.17, 95% CI: 0.76–1.78; 48-hour PR = 1.21, 95% CI: 0.81–1.80). The sensitivity, PPV, and NPV of the H_2_S test was significantly higher when the H_2_S test was incubated for 48 versus 24 hours whereas specificity showed the opposite trend. H_2_S test sensitivity, PPV, and NPV increased with increasing *E. coli* levels, consistent with previous evidence that the H_2_S test is a useful water quality tool in high-contamination settings. However, our results suggest that the H_2_S test is not an effective indicator for waterborne diarrhea.

## INTRODUCTION

Drinking water is an important transmission pathway for diarrheal pathogens.^1,2^ Improving the microbial quality of drinking water by household treatment and safe storage has been shown to reduce diarrhea.^3–5^ In low-resource settings, measuring microbial water quality can be difficult in the absence of accessible, appropriate, and affordable water quality testing methods.^6^ Microbiological water quality is typically assessed using *Escherichia coli* (*E. coli*), an indicator of fecal contamination and waterborne pathogens.^1^ Detection of *E. coli* by membrane filtration requires dedicated facilities and specialized training.^7^ The hydrogen sulfide (H_2_S) presence/absence test is an inexpensive, easy-to-use, and portable alternative field-based water quality test^8^ which has been used globally for more than two decades^9^ and gained popularity as a low-cost assay for assessing fecal contamination.^10^

The H_2_S test is intended to detect bacteria of fecal origin, some of which are able to reduce organic sulfur to sulfide as H_2_S gas. This reacts with the reagents in the test vial to form a black precipitate and allows visual detection of fecal contamination by examining the color of the water in the vial. However, there is concern that the test may also detect bacteria that are not associated with fecal contamination and their attendant pathogens.^11^ In addition, the performance of the H_2_S test as an indicator of waterborne diarrhea risk is contentious. A previous study in India found no association between diarrhea and water quality assessed by H_2_S testing.^7^ However, diarrhea prevalence was very low in this cohort (2.4%), and the study collected water samples concurrently with disease information. This lack of temporality introduces the potential for reverse causation that could bias the observed association between water quality and illness.^12^ When children have diarrhea, they can contaminate the household drinking water by indiscriminate defecation. Alternatively, caregivers may choose to treat a child’s drinking water when the child is ill. The authors indeed found that caregivers in this study were more likely to boil drinking water when the child had diarrhea, cough, or congestion, which could have the biased study findings toward the null.^7^ In addition, lack of time ordering can further weaken the association between water quality and diarrhea because given the temporal variability of household water quality, water contamination measured at the time when illness outcomes have already occurred is an imperfect proxy for the water contamination during the relevant exposure period before disease incubation.

To evaluate the H_2_S test as a drinking water quality management tool, our study aimed to estimate the association between H_2_S test results in stored household drinking water samples and subsequent diarrhea among children < 5 years of age, recorded 1 month after the water quality measurement to establish temporal order. We also assessed the H_2_S test’s sensitivity, specificity, and positive and negative predictive value (PPV, NPV) in detecting fecal contamination in comparison to the standard water quality indicator of *E. coli* enumerated by membrane filtration.

## MATERIALS AND METHODS

### Study setting.

Our study was nested within a randomized controlled trial of the health impact of treating and safely storing shallow tube well drinking water conducted in rural Bangladesh. Details of the study design and population have been reported.^13^ In brief, the trial enrolled 1,800 households that consistently relied on a shallow tube well (< 250 ft) as their primary source of drinking water. Our analysis used measurements from the 600 households enrolled in the control arm of the trial.

### Data collection.

Field staff collected baseline data on household characteristics between July and September 2011, and followed up with households longitudinally between October 2011 and November 2012 approximately once a month, with a total of 10 visits per household. During each follow-up visit, field staff collected stored drinking water samples for H_2_S analysis and detection of *E. coli* by membrane filtration from a systematic subset of enrolled households. Samples for H_2_S testing were collected from the first 50% of households visited each day for each field worker during the first three household visits and from the first 10% during subsequent visits, whereas samples for *E. coli* testing were collected from the first 10% of households visited each day during all visits. Field staff also collected data on reported water treatment practices at the time of follow-up using a structured questionnaire and conducted spot check observations on drinking water containers and household hygiene and sanitation conditions (e.g., observed the availability of hand washing station, observed the presence of latrine in compound).

We analyzed the samples by using the H_2_S test within 8 hours of collection. The NGO Forum, Dhaka, Bangladesh (http://www.ngof.org) supplied H_2_S test vials for use in this evaluation.^9^ The kits use a flattened vial with a screw top cap and a plastic inner cap. All components were sterilized by autoclaving at the NGO Forum. Field workers trained by study investigators on sterile technique added 20 mL of water into the H_2_S vials. During inoculation, the field workers removed the plastic inner cap and replaced it as aseptically as possible. Field supervisors observed sample collection to ensure a sterile technique. The vials were vigorously shaken immediately after inoculation to inhibit the growth of anaerobic and microaerophilic organisms and stored at ambient temperature. Trained microbiologists inspected the vials for color change 24 hours and 48 hours later. The test was interpreted as positive if the color changed from clear to black.

To enumerate *E. coli*, field staff collected approximately 250 mL of water from the household’s primary storage container using a sterile Whirlpak bag (Nasco Modesto, Salida, CA). The samples were transported on ice and analyzed within 8 hours of collection. *Escherichia coli* was enumerated with membrane filtration using U.S. EPA Method 1604^14^; 100 mL aliquots were processed without dilution. Ten per cent blanks and 10% duplicates were processed for quality control. *Escherichia coli* concentration was measured in colony forming units (CFU) per 100 mL, and the samples were classified according to the WHO thresholds of no risk (< 1 CFU/100 mL), low risk (1–10 CFU/100 mL), moderate risk (11–100 CFU/100 mL), and high risk or above (> 100 CFU/100 mL).^1^

At each household visit, the field staff recorded the caregiver-reported diarrhea prevalence in children < 5 years in all households. We defined diarrhea as three or more loose or watery stools in 24 hours.^15,16^ Two different recall periods (2-days and 7-days before the interview) were used to assess the effect of symptom recall on study findings as longer recall periods are more prone to reporting error^17,18^ and could therefore weaken the association between reported diarrhea and water quality.

### Data analysis.

The primary health outcome in this study was caregiver-reported diarrhea among children < 5 years. We matched H_2_S measurements from each follow-up visit with diarrhea measurements collected at the following visit approximately 1 month later. We assessed the association between the H_2_S test (incubated for 24 hours and 48 hours) and diarrhea prevalence (with 2-day and 7-day recall) using generalized estimating equations to estimate prevalence ratios (PRs), with robust standard errors to account for clustering at the household level arising from multiple children in each household and multiple diarrhea measurements for each child. We conducted bivariate and multivariable analyses. We identified potential confounders as characteristics that could be associated with water quality and predictive of diarrhea. In multivariable models, we included all covariates that were associated with diarrhea prevalence at the *P* < 0.2 level in bivariate analyses.

In the subset of 525 water samples with paired H_2_S and *E. coli* measurements, we calculated the sensitivity, specificity, PPV, and NPV for the H_2_S test read at 24 hours and 48 hours compared with *E. coli* detected by membrane filtration, along with the corresponding 95% exact confidence intervals (CIs).We conducted all statistical analyses using STATA software (version 13).

### Ethical considerations.

All households provided written informed consent. The randomized controlled trial that our analysis was nested in was registered at ClinicalTrials.gov (NCT01350063).The trial protocol was reviewed and approved by human subjects review committees at the International Center for Diarrheal Disease Research, Bangladesh (icddr,b) (PR-10038) and the University of California, Berkeley (2010-05-1630).

## RESULTS

A total of 1,157 H_2_S samples were collected from 600 households over 10 visits. Hydrogen sulfide data from the last household visit were excluded because of no subsequent health data, yielding 1,105 samples from nine visits for analysis. With an average of 1.2 children < 5 years per household, this yielded 1,348 paired H_2_S and diarrhea measurements.

The caregiver-reported prevalence of diarrhea among children < 5 years was 8% for a 2-day recall window and 11% for a 7-day recall window (Table 1). Of the 1,348 H_2_S samples, 28% (383) were positive after 24 hours and 70% (949) after 48 hours of incubation, whereas *E. coli* was detected by membrane filtration in 90% (470/525) of samples. The geometric mean *E. coli* count was 1.2 CFU per 100 mL (SD = 0.84) (Table 1). The most frequently observed water storage containers were *kalash* (a lidless aluminum vessel with a narrow mouth but a wide brim that is typically covered using a plate) (73%) and pitchers (a wide-mouth plastic or metal container that can have a tight-fitting lid or be covered using a plate) (24%). Among these, 45% of the *kalash* and 16% of the pitchers were observed to be covered. Two per cent of respondents reported treating their drinking water (Table 1).

**Table 1:** Child and household characteristics among enrolled children < 5 years in rural Bangladesh (*N* = 1348)

	*N*	*n* (%)
Child characteristics		
Age at enrolment in months, mean (SD)	1,348	12 (3.1)
Female	1,348	639 (47)
Currently breastfeeding	1,348	1,302 (97)
2-day prevalence of diarrhea	1,348	112 (8.3)
7-day prevalence of diarrhea	1,348	151 (11)
Household characteristics		
Respondent’s age in years, mean (SD)	584	26 (5.6)
Respondent’s education		
No education	584	165 (28)
Primary	584	191 (33)
Secondary and above	584	228 (39)
Number of persons per household,mean (SD)	584	5.3 (1.97)
Number of rooms in household,mean (SD)	584	1.6 (0.97)
Monthly household income (USD),mean (SD)	573	95 (78)
Households with:		
Natural wall (made by jute/bamboo/mud)	584	197 (34)
Electricity	584	197 (34)
Cell phone	584	397 (68)
TV	584	131 (22)
Household has access to latrine	584	484 (83)
Latrine type		
Improved sanitation facility*	484	186 (38)
Unimproved sanitation facility†	484	298 (62)
Households with:		
HWS	584	466 (80)
HWS < 10 steps from latrine	466	183 (39)
HWS with water	466	419 (90)
HWS with soap	466	188 (40)
Drinking water storage container and covering status		
*Kalash* (narrow-mouth container)		
Covered	428	192 (45)
Uncovered	428	236 (55)
Pitcher (wide-mouth container)		
Covered	137	21 (16)
Uncovered	137	116 (84)
Household treats drinking water	584	13 (2.2)
Household stored water quality		
Stored water samples were H_2_S-positive		
With 24-hr incubation	1,348	383 (28)
With 48-hr incubation	1,348	949 (70)
*Escherichia coli* was detected by membrane filtration in stored water samples	525	470 (90)
*Escherichia coli* count (CFU per 100 mL)in stored water samples, geometric mean (SD)	525	1.2 (0.84)

CFU = colony forming units; HWS = hand washing station; SD = standard deviation;USD = US dollars.

* Improved facilities include flush/pour flush latrines that drain to piped sewer, septic tank, or off-set pit; pit latrines with slab and water seal or with slab, no water seal but lid; and composting toilets.

† Unimproved facilities include flush/pour flush latrines that drain into the environment; open pits; pit latrines without slab; pit latrines with slab but no water seal and no lid; and hanging toilets.

In bivariate analyses, there was no association between H_2_S-positive water samples at 24 or 48 hours and 2-day or 7-day child diarrhea prevalence (Table 2). In multivariable analyses controlling for household water, sanitation and hygiene conditions (e.g., reported water treatment practices, observed the presence of latrine in compound, observed the availability of hand washing station) and household wealth (all *P* < 0.2 in bivariate analysis with diarrhea), H_2_S-positive water, with 24 hours or 48 hours of incubation, was not associated with 2-day child diarrhea prevalence (24-hour PR = 1.03, 95% CI: 0.63–1.69; 48-hour PR = 0.89, 95% CI: 0.58–1.38; Table 2) or 7-day diarrhea prevalence (24-hour PR = 1.17, 95% CI: 0.76–1.78; 48-hour PR = 1.21, 95% CI: 0.81–1.80; Table 2).

**Table 2:** Association between hydrogen sulfide test in stored drinking water and diarrhea among children < 5 years of age measured over 1 year in rural Bangladesh (*N* = 1,348)

	*N*	Diarrhea *n* (%)	Unadjusted* PR (95% CI)	*P* value	Adjusted† PR (95% CI)	*P* value
2-day prevalence of diarrhea						
H_2_S test with 24-hour incubation						
Positive	383	27 (7.05)	0.79 (0.51, 1.22)	0.29	1.03 (0.63, 1.69)	0.99
Negative	965	85 (8.81)	Ref	–	Ref	–
H_2_S test with 48-hour incubation						
Positive	949	75 (7.90)	0.84 (0.57, 1.24)	0.38	0.89 (0.58, 1.38)	0.54
Negative	399	37 (9.27)	Ref	–	Ref	–
7-day prevalence of diarrhea						
H_2_S test with 24-hour incubation						
Positive	383	41 (11)	0.92 (0.64, 1.32)	0.66	1.17 (0.76, 1.78)	0.55
Negative	965	110 (12)	Ref	–	Ref	–
H_2_S test with 48-hour incubation						
Positive	949	110 (12)	1.12 (0.78, 1.60)	0.54	1.21 (0.81, 1.80)	0.40
Negative	399	41 (10)	Ref	–	Ref	–

CI = confidence interval; H_2_S = hydrogen sulfide; PR = prevalence ratio.

* We determined the prevalence ratio by using generalized estimating equation to adjust for multiple samples and children per household.

† Adjusted for child age, wealth index, mother’s education, season, access to latrine, presence of hand washing station with water and soap.

When we compared the H_2_S test with detection of any *E. coli* by membrane filtration, the sensitivity of the H_2_S test increased significantly with incubation time from 47% (42–52%) at 24 hours to 83% (79–86%) at 48 hours, whereas specificity showed the opposite trend, decreasing from 85% (73–94%) at 24 hours to 49% (35–63%) at 48 hours (Table 3). PPV and NPV were also significantly higher with 48-hours versus 24-hours incubation. The H_2_S test sensitivity, PPV, and NPV increased with increasing level of *E. coli* contamination (Table 3).

**Table 3:** Sensitivity, specificity, positive predictive value and negative predictive value of H_2_S test against *Escherichia coli* by membrane filtration, for different *Escherichia coli* risk categories in stored household water samples in rural Bangladesh (*N* = 525)

Duration of H_2_S test incubation (hour)	*Escherichia coli* level (CFU/100 mL) by membrane filtration	*N*	Number of H_2_S positive samples	Number of H_2_S negative samples	Sensitivity % (95% exact CI)	Specificity % (95% exact CI)	PPV % (95% exact CI)	NPV % (95% exact CI)
24 hours	No risk (< 1)	55	8	47	–	85 (73, 94)	–	–
	Low risk (1–10)	144	37	107	26 (19, 34)		82 (68, 92)	31 (23, 38)
	Moderate risk (11–100)	208	94	114	45 (39, 52)		92 (85, 97)	29 (22, 37)
	High risk or above (> 100)	107	81	26	76 (67, 84)		91 (83, 96)	64 (52, 75)
	All positive (≥ 1)*	470	221	249	47 (42, 52)		97 (93, 98)	16 (12, 21)
48 hours	No risk (< 1)	55	28	27	–	49 (35, 63)	–	–
	Low risk (1–10)	144	104	40	72 (65,80)		79 (71, 85)	40 (28, 53)
	Moderate risk (11–100)	208	175	33	84 (79, 89)		86 (81, 91)	45 (32, 58)
	High risk or above (> 100)	107	99	8	93 (87, 98)		78 (70, 84)	77 (60, 90)
	All positive (≥ 1)*	470	388	82	83 (79, 86)		93 (90, 95)	25 (17, 34)

CFU = colony forming units; CI = confidence interval; H_2_S = hydrogen sulfide; NPV = negative predictive value; PPV = positive predictive value.

* The sum of the sample numbers in the low, moderate and high risk categories is smaller than the total number of positive samples because of 11 confluent (positive but not countable) samples.

## DISCUSSION

In our study, we found no association between the H_2_S test in stored drinking water and diarrhea among children < 5 years of age, despite establishing temporal order by measuring diarrhea prevalence approximately 1 month after collecting the water samples. Microbial water quality indicators are often poor surrogates for the actual health risks associated with drinking water.^19–21^ Previous systematic reviews and meta-analyses found conflicting evidence on the association between diarrhea and *E. coli* and fecal coliforms in drinking water as indicators of drinking water contamination.^19,22^ A previous study in India also found no association between diarrhea and water quality measured by the H_2_S test.^7^ However, these studies had problems with exposure-disease temporality because of simultaneous measurements of water quality and disease outcomes.

Our findings contrast with a separate analysis of *E. coli* data from the same parent trial^12^ as well as a different study, also conducted in rural Bangladesh,^23^ both of which demonstrated a positive association between drinking water *E. coli* and subsequent diarrhea. The separate analysis of *E. coli* data from our study dataset found that, for each log10 increase in *E. coli* in drinking water, diarrhea prevalence measured approximately 1 month later increased by 50%.^12^ In the other study, children whose drinking water contained *E. coli* were found to have 35% higher diarrhea prevalence, measured 3–46 days after the water quality assessment.^23^ Taken together, this evidence suggests that in this context, the H_2_S test did not accurately signal the presence of waterborne pathogens that caused diarrhea, whereas *E. coli* levels did correspond to diarrhea risk.

One potential limitation of the H_2_S test is that its simple operation allows unskilled personnel to carry out the procedure, potentially leading to problems with sterile technique and interpretation of test results. Sample collection in our study was conducted by field staff with a minimum of college-level education and additional training on sterile technique by study investigators. Blank samples collected for quality control showed no evidence of background contamination. The interpretation of tests was performed by microbiologists trained at the master’s level. The lack of association between H_2_S test results and diarrhea outcomes is therefore unlikely to be due to errors in the execution of the text; other low-income country settings where the H_2_S test is routinely performed by unskilled staff may encounter further problems with test performance.

One of the limitations of our study was that there was an approximately 1-month gap between water quality and diarrhea measurement, which is longer than the incubation period for most bacterial and viral fecal pathogens that cause diarrhea.^12^ A shorter gap between water quality and diarrhea measurements that reflects the incubation period of bacteria and viruses could potentially demonstrate an association between the H_2_S test in drinking water and subsequent diarrhea. However, the aforementioned studies in Bangladesh that demonstrated a clear link between *E. coli* and diarrhea used a similarly long gap, so the duration between the measurements is unlikely to explain the lack of association between the H_2_S test and subsequent diarrhea.

Another limitation was that our water samples were one-time grab samples. It is possible that these do not fully describe household water quality, which varies significantly over short time frames. That is, a one-time positive or negative H_2_S test might not be an accurate representation of the overall quality of water consumed by children in the household.^24,25^ A study in India that collected repeated H_2_S samples showed that the per cent of H_2_S-positive samples was linearly related to the log10 total coliform concentration.^7^ Repeated H_2_S tests may therefore be a more accurate water quality indicator than a single test.

It is also possible that our study was conducted at a time of relatively good drinking water quality and diarrhea was predominantly transmitted by non-waterborne routes. However, the randomized trial that this work was nested within found evidence of *E. coli* contamination in 89% of stored drinking water samples in the control arm and 31–36% reduction in diarrhea in the water treatment and safe storage arms, indicating waterborne transmission.^13^

We collected caregiver-reported diarrhea prevalence and for any self-reported, subjective outcome, there is a potential for differential reporting relative to exposure status.^17,18^ Participants included in our analysis (from the control arm of the parent trial) received no water treatment intervention and were not aware of their microbiological water quality. Misreporting of outcomes was therefore likely to be nondifferential by water quality and bias the observed association toward the null. Moreover, our findings were similar for 2-day versus 7-day diarrhea recall periods even though the longer window is more susceptible to recall error, suggesting that inaccurate recall is unlikely to explain the lack of association between diarrhea and water quality measured by H_2_S.

We also note that our results reflect the conditions in rural Bangladesh and may not be generalizable to other contexts as the H_2_S test may perform differently in different settings that host different bacterial ecologies. Indeed, a systematic review and meta-analysis found wide variation in the diagnostic accuracy of the H_2_S test.^26^

We found that the sensitivity, PPV, and NPV of the H_2_S test increased, and specificity decreased with increasing incubation time, consistent with prior evidence.^9,26^ These results suggest using the H_2_S test with a 48-hour incubation period in settings where high sensitivity is preferred. H_2_S test sensitivity, PPV, and NPV increased with increasing levels of *E. coli* contamination, suggesting that both tests measure related characteristics and confirming previous evidence that the H_2_S test 3maybe a useful tool to measure water quality in high-contamination settings.^26^ However, the lack of an association between drinking water quality measured by the H_2_S test and subsequent diarrhea indicates that in our study setting, the H_2_S test is not an effective water quality indicator for assessing the risk for diarrhea.
